# Supplementary material for: Epidemiology and burden of multidrug-resistant bacterial infection in a developing country
Source: eLife. 2016 Sep 6;5:e18082. doi: 10.7554/eLife.18082 (PMC5030096; doi:10.7554/eLife.18082)
Supplement: Supplementary file 1. — DOI: http://dx.doi.org/10.7554/eLife.18082.019 [file elife-18082-supp1.docx]

- **Supplementary file 1. Factors associated with 30-day mortality of bacteremia patients.**

| **Variables** | **CAB** |  | **HCAB** |  | **HAB** |  |
| --- | --- | --- | --- | --- | --- | --- |
|  | **aOR (95% CI)** | **P value** | **aOR (95% CI)** | **P value** | **aOR (95% CI)** | **P value** |
| **Age (year)** |  |  |  |  |  |  |
| **≥65** | 1.0 | <0.001 | 1.0 | <0.001 | 1.0 | <0.001 |
| **45-64** | 1.0 (0.9-1.1) |  | 0.8 (0.6-1.0) |  | 0.7 (0.6-0.9) |  |
| **16-44** | 0.7 (0.6-0.9) |  | 0.8 (0.5-1.1) |  | 0.5 (0.4-0.7) |  |
| **1-15** | 0.2 (0.1-0.3) |  | 0.5 (0.2-0.9) |  | 0.3 (0.2-0.4) |  |
| **<1** | 0.3 (0.2-0.4) |  | 0.1 (0.04-0.4) |  | 0.3 (0.2-0.4) |  |
| **Gender** |  |  |  |  |  |  |
| **Male** | 1.5 (1.4-1.7) | <0.001 | 1.0 (0.8-1.3) | 0.80 | 0.9 (0.8-1.1) | 0.51 |
| **Admission year** |  |  |  |  |  |  |
| **2004** | 1.0 | 0.04 | 1.0 | 0.74 | 1.0 | 0.13 |
| **2005** | 0.9 (0.6-1.2) |  | 0.8 (0.4-1.7) |  | 0.7 (0.4-1.2) |  |
| **2006** | 0.7 (0.5-1.0) |  | 1.2 (0.6-2.4) |  | 0.6 (0.4-1.0) |  |
| **2007** | 1.1 (0.8-1.4) |  | 0.8 (0.4-1.6) |  | 1.0 (0.6-1.5) |  |
| **2008** | 1.1 (0.8-1.5) |  | 0.9 (0.5-1.7) |  | 0.7 (0.4-1.0) |  |
| **2009** | 1.0 (0.8-1.3) |  | 0.8 (0.4-1.4) |  | 0.7 (0.5-1.1) |  |
| **2010** | 1.0 (0.8-1.3) |  | 0.8 (0.4-1.4) |  | 0.9 (0.6-1.4) |  |
| **Time to bacteraemia** |  |  |  |  |  |  |
| **3-7 days** | - | - | - | - | 1.0 | 0.63 |
| **>7-14 days** |  |  |  |  | 1.1 (0.9-1.3) |  |
| **>14-30 days** |  |  |  |  | 1.0 (0.7-1.2) |  |
| **>30 days** |  |  |  |  | 1.2 (0.9-1.6) |  |

NOTE: CAB=Community-acquired bacteraemia, HCAB=Healthcare-associated bacteraemia, HAB= Hospital-acquired bacteraemia, and aOR=adjusted odds ratios. Time to bacteremia was defined as the duration between hospital admission and the date positive blood culture was taken. Multivariable logistic regression models were used and applied to each group of patients (CAB, HCAB and HAB) separately. Variables in the models also included organisms, MDR and the interaction variable between organisms and MDR. Association between MDR of each organism and mortality is shown in Figure 2.
