# Supplementary material for: Epidemiology and burden of multidrug-resistant bacterial infection in a developing country
Source: eLife. 2016 Sep 6;5:e18082. doi: 10.7554/eLife.18082 (PMC5030096; doi:10.7554/eLife.18082)
Supplement: Supplementary file 2. — DOI: http://dx.doi.org/10.7554/eLife.18082.020 [file elife-18082-supp2.docx]

- **Supplementary file 2. Parameters used to estimate mortality attributable to multidrug-resistance in Thailand**

| - Parameter | - Unit | - Value | - Reference |
| --- | --- | --- | --- |
| - Cumulative incidence of MDR *S. aureus* bacteraemia in Thailand in 2010 | - Patients | - 2,017 | - Pumart, 2012.[^15^](#_ENREF_23) |
| - Cumulative incidence of MDR *S. aureus* LRTI in Thailand in 2010 | - Patients | - 13,683 | - Pumart, 2012.[^15^](#_ENREF_23) |
| - Cumulative incidence of MDR *S. aureus* UTI in Thailand in 2010 | - Patients | - 288 | - Pumart, 2012.[^15^](#_ENREF_23) |
| - Cumulative incidence of MDR *S. aureus* SSTI in Thailand in 2010 | - Patients | - 864 | - Pumart, 2012.[^15^](#_ENREF_23) |
| - Cumulative incidence of MDR *S. aureus* other site in Thailand in 2010 | - Patients | - 1,873 | - Pumart, 2012.[^15^](#_ENREF_23) |
| - Attributable mortality of MDR *S. aureus* bacteraemia | - % | - 14.8 | - This study |
| - Attributable mortality of MDR *S. aureus* LRTI | - % | - 17.5 | - This study and Martone, 1998.[^30^](#_ENREF_24) |
| - Attributable mortality of MDR *S. aureus* UTI | - % | - 1.2 | - This study and Martone, 1998.[^30^](#_ENREF_24) |
| - Attributable mortality of MDR *S. aureus* SSTI | - % | - 3.3 | - This study and Martone, 1998.[^30^](#_ENREF_24) |
| - Attributable mortality of MDR *S. aureus* other site | - % | - 4.3 | - This study and Martone, 1998.[^30^](#_ENREF_24) |
| - Cumulative incidence of MDR *E. coli* bacteraemia in Thailand in 2010 | - Patients | - 144 | - Pumart, 2012.[^15^](#_ENREF_23) |
| - Cumulative incidence of MDR *E. coli* LRTI in Thailand in 2010 | - Patients | - 1,728 | - Pumart, 2012.[^15^](#_ENREF_23) |
| - Cumulative incidence of MDR *E. coli* UTI in Thailand in 2010 | - Patients | - 2,449 | - Pumart, 2012.[^15^](#_ENREF_23) |
| - Cumulative incidence of MDR *E. coli* SSTI in Thailand in 2010 | - Patients | - 346 | - Pumart, 2012.[^15^](#_ENREF_23) |
| - Cumulative incidence of MDR *E. coli* other site in Thailand in 2010 | - Patients | 6,449 | - Pumart, 2012.[^15^](#_ENREF_23) |
| - Attributable mortality of MDR *E. coli* bacteraemia | - % | - 13.8 | - This study |
| - Attributable mortality of MDR *E. coli* LRTI | - % | - 16.3 | - This study and Martone, 1998.[^30^](#_ENREF_24) |
| - Attributable mortality of MDR *E. coli* UTI | - % | - 1.2 | - This study and Martone, 1998.[^30^](#_ENREF_24) |
| - Attributable mortality of MDR *E. coli* SSTI | - % | - 3.1 | - This study and Martone, 1998.[^30^](#_ENREF_24) |
| - Attributable mortality of MDR *E. coli* other site | - % | - 4.0 | - This study and Martone, 1998.[^30^](#_ENREF_24) |
| - Cumulative incidence of MDR *K. pneumoniae* bacteraemia in Thailand in 2010 | - Patients | - 288 | - Pumart, 2012.[^15^](#_ENREF_23) |
| - Cumulative incidence of MDR *K. pneumoniae* LRTI in Thailand in 2010 | - Patients | - 8,930 | - Pumart, 2012.[^15^](#_ENREF_23) |
| - Cumulative incidence of MDR *K. pneumoniae* UTI in Thailand in 2010 | - Patients | - 1,728 | - Pumart, 2012.[^15^](#_ENREF_23) |
| - Cumulative incidence of MDR *K. pneumoniae* SSTI in Thailand in 2010 | - Patients | - 115 | - Pumart, 2012.[^15^](#_ENREF_23) |
| - Cumulative incidence of MDR *K. pneumoniae* other site in Thailand in 2010 | - Patients | - 4,178 | - Pumart, 2012.[^15^](#_ENREF_23) |
| - Attributable mortality of MDR *K. pneumoniae* bacteraemia | - % | - 2.4 | - This study |
| - Attributable mortality of MDR *K. pneumoniae* LRTI | - % | - 2.8 | - This study and Martone, 1998.[^30^](#_ENREF_24) |
| - Attributable mortality of MDR *K. pneumoniae* UTI | - % | - 0.2 | - This study and Martone, 1998.[^30^](#_ENREF_24) |
| - Attributable mortality of MDR *K. pneumoniae* SSTI | - % | - 0.5 | - This study and Martone, 1998.[^30^](#_ENREF_24) |
| - Attributable mortality of MDR *K. pneumoniae other site* | - % | - 0.7 | - This study and Martone, 1998.[^30^](#_ENREF_24) |
| - Cumulative incidence of MDR *P. aeruginosa* bacteraemia in Thailand in 2010 | - Patients | - 94 | - Pumart, 2012.[^15^](#_ENREF_23) |
| - Cumulative incidence of MDR *P. aeruginosa* LRTI in Thailand in 2010 | - Patients | - 4,897 | - Pumart, 2012.[^15^](#_ENREF_23) |
| - Cumulative incidence of MDR *P. aeruginosa* UTI in Thailand in 2010 | - Patients | - 169 | - Pumart, 2012.[^15^](#_ENREF_23) |
| - Cumulative incidence of MDR *P. aeruginosa* SSTI in Thailand in 2010 | - Patients | - 94 | - Pumart, 2012.[^15^](#_ENREF_23) |
| - Cumulative incidence of MDR *P. aeruginosa* other site in Thailand in 2010 | - Patients | - 864 | - Pumart, 2012.[^15^](#_ENREF_23) |
| - Attributable mortality of MDR *P. aeruginosa* bacteraemia | - % | - 4.4 | - This study |
| - Attributable mortality of MDR *P. aeruginosa* LRTI | - % | - 5.2 | - This study and Martone, 1998.[^30^](#_ENREF_24) |
| - Attributable mortality of MDR *P. aeruginosa* UTI | - % | - 0.4 | - This study and Martone, 1998.[^30^](#_ENREF_24) |
| - Attributable mortality of MDR *P. aeruginosa* SSTI | - % | - 1.0 | - This study and Martone, 1998.[^30^](#_ENREF_24) |
| - Attributable mortality of MDR *P. aeruginosa* other site | - % | - 1.3 | - This study and Martone, 1998.[^30^](#_ENREF_24) |
| - Cumulative incidence of MDR *Acinetobacter* spp bactereamia in Thailand in 2010 | - Patients | - 864 | - Pumart, 2012.[^15^](#_ENREF_23) |
| - Cumulative incidence of MDR *Acinetobacter* spp LRTI in Thailand in 2010 | - Patients | - 29,672 | - Pumart, 2012.[^15^](#_ENREF_23) |
| - Cumulative incidence of MDR *Acinetobacter* spp UTI in Thailand in 2010 | - Patients | - 1,152 | - Pumart, 2012.[^15^](#_ENREF_23) |
| - Cumulative incidence of MDR *Acinetobacter* spp SSTI in Thailand in 2010 | - Patients | - 432 | - Pumart, 2012.[^15^](#_ENREF_23) |
| - Cumulative incidence of MDR *Acinetobacter* spp other site in Thailand in 2010 | - Patients | - 4,433 | - Pumart, 2012.[^15^](#_ENREF_23) |
| - Attributable mortality of MDR *Acinetobacter* spp bacteraemia | - % | - 40.6 | - This study |
| - Attributable mortality of MDR *Acinetobacter* spp LRTI | - % | - 47.9 | - This study and Martone, 1998.[^30^](#_ENREF_24) |
| - Attributable mortality of MDR *Acinetobacter* spp UTI | - % | - 3.4 | - This study and Martone, 1998.[^30^](#_ENREF_24) |
| - Attributable mortality of MDR *Acinetobacter* spp SSTI | - % | - 9.0 | - This study and Martone, 1998.[^30^](#_ENREF_24) |
| - Attributable mortality of MDR *Acinetobacter* spp other site | - % | - 11.7 | - This study and Martone, 1998.[^30^](#_ENREF_24) |

- LRTI = Lower respiratory track infection, UTI = Urinary tract infection, SSTI = Skin and Soft tissue infection
